# Radical Prostatectomy and Intraoperative Radiation Therapy in High-Risk Prostate Cancer

**DOI:** 10.1155/2012/687230

**Published:** 2012-01-30

**Authors:** Giansilvio Marchioro, Alessandro Volpe, Roberto Tarabuzzi, Giuseppina Apicella, Marco Krengli, Carlo Terrone

**Affiliations:** ^1^Division of Urology, University of Eastern Piedmont, Maggiore della Carità Hospital, Novara, Italy; ^2^Division of Radiotherapy, University of Eastern Piedmont, Maggiore della Carità Hospital, Novara, Italy

## Abstract

Intraoperative electron beam radiotherapy (IOERT) for prostate cancer (PC) is a radiotherapeutic technique, giving high doses of radiation during radical prostatectomy (RP). This paper presents the published treatment approaches for intraoperative radiotherapy analyzing functional outcome, morbidity, and oncological outcome in patients with clinical intermediate-high-risk prostate cancer. A systematic review of the literature was performed, searching PubMed and Web of Science. A “free text” protocol using the term intraoperative radiotherapy and prostate cancer was applied. Ten records were retrieved and analyzed including more than 150 prostate cancer patients treated with IOERT. IOERT represents a feasible technique with acceptable surgical time and minimal toxicity. A greater number of cases and longer follow-up time are needed in order to assess the long-term side effects and oncological outcome.

## 1. Introduction

The optimal treatment of locally advanced PC is still unclear [1]. The use of radical prostatectomy (RP) alone is controversial, and external beam radiation (EBRT) associated with hormonal therapy (HT) has been the traditional treatment modality for this stage of disease [2]. However, even with the use of multimodal approaches, only a 37–62% and 44% disease-free survival at 5 and 10 years can be obtained [3–7] and side effects of these treatments are not limited [2].

With the aim to improve the clinical outcomes of locally advanced PC, various radiotherapeutic approaches have been implemented. IOERT is being investigated as a technique to deliver a high dose of radiation to a locally advanced tumor protecting adjacent normal tissues at the time of surgery. This new technique has been used for treatment of several tumours as a boost or sole radiation treatment before or after tumour resection with the aim to improve local tumour control. Different approaches and different accelerators have been used, as reported in technical and dose-finding studies.

IOERT for PC was first proposed in Japan more than twenty years ago, either as a single treatment [8, 9] or combined with pelvic lymphadenectomy (PLND) or EBRT to pelvic lymph nodes [10, 11]. Recently, three Italian centers have reported series of intermediate and high-risk PC patients treated with IOERT combined with RP and PLND [12–14]. The potential advantage of IOERT is to allow optimal targeting and identification of the prostate and surrounding structures. Recent radiobiological studies suggest that the use of a single high fraction of radiation may increase the efficacy of the treatment leading to higher tumor cell killing [15].

 The aim of this paper is to describe the different technical approaches of IOERT and the available results in terms of clinical outcome for locally advanced PC.

## 2. Material and Methods

A literature search was performed using PubMed and Web of Science from 1975 to 2011. The keywords IOERT and PC were used. A free-text strategy was applied without limitations. We retrieved 11 references dealing with IOERT and PC (Table 1). Only phase I-II studies are available. No randomized clinical trials (RCTs), systematic reviews of cohort studies, and low-quality RCTs are reported. The aim was not to produce a meta-analysis but to critically evaluate and discuss the use of IOERT in the treatment of PC.

## 3. Result

The first series of IOERT for PC was reported by the Kyoto University and Saitama Cancer Center in Japan. The authors initially carried out IOERT as single treatment or in combination with PLND or EBRT to pelvic lymph nodes [9–11, 16, 17]. Perineal approach without RP using electron energy from 10 to 14 Mev has been performed in 14 patients by Takahashi et al. [9]. Five patients treated by IOERT alone received single doses of 2800 to 3500 cGy. Two patients treated with 2800 and 3000 cGy, respectively, had local recurrence. A single dose of 2000 or 2500 cGy was delivered intraoperatively to 9 patients as a boost dose in conjunction with external irradiation of 5000 cGy for the treatment of pelvic lymph nodes. All these patients achieved local control. No patients in the overall series developed serious bladder, urethral, or rectal complications. An update from the same center reported a local control and 5-year survival rates of 81% and 72%, respectively, with 2% late toxicity consisting of chronic cystitis and urethral stricture [17].

The experience of the Saitama Cancer Center began with the perineal approach and switched to the retropubic approach after the first 10 cases, due to the potential risk of rectal damage, impossible PNLD, and patient discomfort [10, 11]. Radiation therapy included 25–30 Gy of IOERT on the prostate and 30 Gy of external beam radiotherapy to the small pelvic region. Most patients received additional androgen ablation treatment. The authors reported 92% and 87% overall survival rates in 35 patients with stage B and stage C disease, respectively, without severe side effects.

More recently, Italian authors reported phase I-II studies with a relatively higher number of patients compared with the Japanese series [12–14, 18, 19]. 

A different treatment approach was adopted by three Italian centers. In Saracino's series, 34 patients with localized PC with only one high-risk factor (Gleason score ≥ 7, clinical stage ≥ T2c, or prostate-specific antigen of 11–20 ng/mL) and without clinical evidence of lymph node metastases were treated with RP and IOERT on the tumor bed. Dose levels of 16, 18, and 20 Gy were selected [12]. The IOERT procedure was performed after prostate removal and at the end of bladder-urethral anastomosis. Negative frozen section of bilateral obturator nodes was mandatory. In vivo dosimetry was performed by MOSFET dosimeters inserted in rectal and urethral catheters in order to obtain a reliable dosimetry at the level of the bladder-urethral anastomosis [18].

After a median follow-up of 41 months, the authors reported a local control rate of 91% with biochemical-failure-free survival at 3 years of 77%. They did not observe any relevant early or late toxicity. In this series, unfavorable prognostic factors were stage >T3, PSA > 10 ng/mL at univariate analysis, and surgical positive margins at both univariate and multivariate analyses. Of note, postsurgical T2 stage was detected in 53% of cases [12]. 

**Figure 1:**
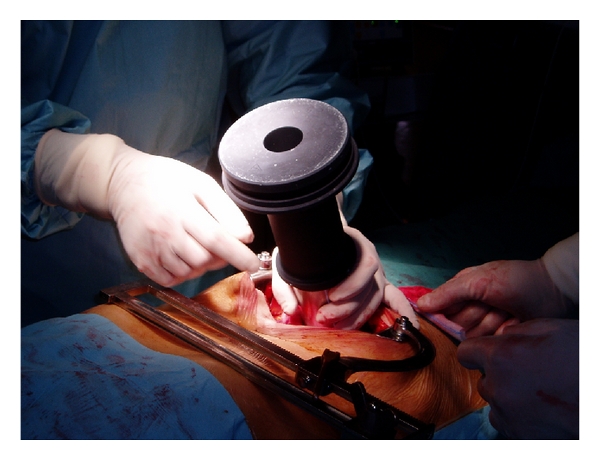
The appropriate collimator is placed and beam energy is chosen in order to include the prostate gland and the surrounding soft tissues with a suitable margin of 0.5–1 cm.

**Figure 2:**
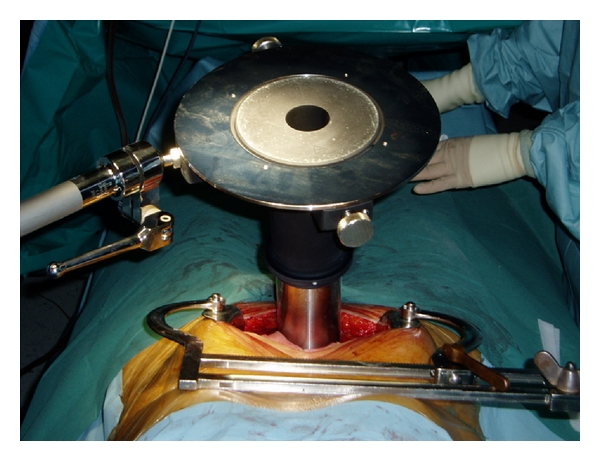
The collimator is fixed to the operating table.

**Figure 3:**
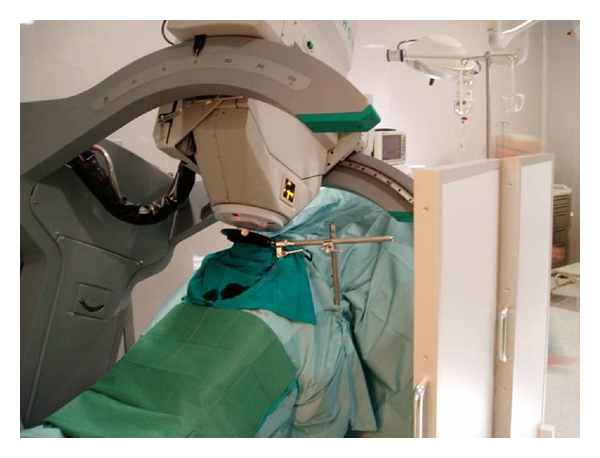
Dedicated linear accelerator (Mobetron, Intraop, Sunnyvale, CA) installed in the operating room.

Orecchia et al. and Krengli et al. reported on 11 and 38 patients, respectively, treated in a similar fashion as in Saracino's study but before prostate removal. In these series, IOERT was not used as a single radiation treatment modality but as an anticipated boost followed by postoperative EBRT according to the pathological findings. A dose of 10–12 Gy was prescribed to the 90% isodose using 9–12 MeV IOERT [13, 14]. In these two series, surgery is performed with a median abdominal incision to approach the retropubic space. The pelvic fascia is prepared and the anterior face of the prostate exposed. Puboprostatic ligaments are sectioned and the deep dorsal vein plexus controlled. The apex of the prostate and the endopelvic urethra are visualized. A stitch is placed as a marker of the bladder neck. The anterior-posterior prostate diameter and the distance from prostate surface to the anterior rectal wall are measured by intraoperative ultrasound. Based on clinical and ultrasound parameters, the appropriate collimator and beam energy are chosen in order to include the prostate gland and the surrounding soft tissues with a suitable margin of 0.5–1 cm Figures 1 and 2. Orecchia et al. administered IORT using a Liac (Info and Tech, Rome, Italy) mobile linear accelerator, while Krengli et al. used a dedicated linear accelerator (Mobetron, Intraop, Sunnyvale, CA) installed in the operating room, delivering electron beams of 9 to 12 MeV for a total dose of 12 Gy (Figure 3). Rectal dose was measured “in vivo” by radio-chromic films placed on the surface of a rectal probe. Three-dimensional conformal RT was delivered 3 months after surgery using 4 to 6 customized beams for a total dose of 46–50 Gy in 25 fractions (2 Gy/fraction) in case of extracapsular extension and/or positive surgical margins at pathology. Adjuvant HT was recommended for 2 years in presence of pT3b-T4 disease or positive lymph nodes (LN+). In case of biochemical failure permanent HT was given [14].

 In 2009 Rocco et al. reported an update of their series, comparing in a matched-pair analysis 33 high-risk patients treated by IOERT with a historical group of 100 patients who underwent RP and adjuvant RT and HT [19]. After a median follow-up of 16 months, only 1 of 33 patients experienced biochemical failure. Surgical outcome was equivalent in the two groups, whereas the urinary continence rate was lightly worse in the IOERT group. However, the continence improved similarly over time in both patient groups. Postsurgical T2 stage was detected in 36% of cases, while most cases were classified as pT3 [19]. 

The series by Krengli et al. was updated in terms of patient number and clinical outcome in 2010 [20]. After a median follow-up of 24 months, all patients were alive and 18% experienced biochemical failure with median time to progression of 27 months (range 6–44). Toxicity and surgical complication rates were low. Complications mainly consisted of lymphoceles (16%) and pelvic hematomas (6%). Eighty-four percent of patients were fully continent, and no grade 3-4 late toxicity was observed. Postsurgical T2 stage was detected in 37% of cases, and most cases were pT3. 

## 4. Discussion

Optimal treatment strategy for locally advanced PC remains unknown. Local control after RP depends on Gleason score, preoperative PSA level, pathological stage, and margins status [21]. 

Multimodal approach which includes adjuvant HT or RT after RP clearly improves the outcomes in men with locally advanced PC [22, 23]. The rationale of IOERT in locally advanced PC is based on the unsatisfactory results obtained by other treatment modalities [24]. Using IOERT, it is possible to irradiate the whole surgical bed, including the tissues surrounding the prostate with a limited dose to the rectum.

IOERT dose of 12 Gy at the 90% isodose compared to doses delivered with conventional EBRT fractionation is similar to the normalized dose of 56.2 Gy with an alpha/beta ratio of 1.5 Gy. The mean dose delivered to the prostate bed of 8.7 Gy reported by Orecchia et al. [13] corresponds to 25.4 Gy with a conventionally EBRT fractionated regimen. Such dose combined with the further 45–50 Gy delivered postoperatively would reach a total dose of 70–75 Gy.

In the Japanese series, patients were treated without RP, with a potential risk of local recurrence.

The techniques used in the Italian studies are different. Orecchia et al. and Krengli et al. reported complete prostate removal after IOERT, while Saracino et al. carried out IOERT after retropubic RP. The first approach aims to optimize the irradiated volume including prostate and surrounding tissues possibly infiltrated by tumor cells. It allows an optimal placement of the most appropriate collimator that can vary in size and bevel angle [14]. Ultrasound measurements of prostate diameter and distance from the rectal wall can help in the choice of the most appropriate beam energy and allows addition of bolus material to modify and optimize the distribution in depth of the radiation dose when needed. Using this technique, the dose to the rectum can be limited because of the interposition of prostate tissue. Finally, this approach can potentially achieve a better irradiation of the prostatic apex, which is frequently a site of recurrence. An important point to underline for any technical approach is the need of precise documentation in terms of quality assurance, such as “in vivo” rectal dosimetry and possibly urethral dosimetry.

Different from the Japanese old experience that delivered a relatively high single dose of 28–35 Gy or of 20–25 Gy when combined with EBRT to the target, Saracino et al. used a single dose up to 22 Gy in intermediate-risk patients, while the other Italian authors used a more conservative approach delivering only part of the dose by IOERT (12 Gy) and adding EBRT in patients with positive margins or extracapsular disease.

A potential critical aspect of this approach is the time interval between IOERT and EBRT, that is, about 2-3 months. The rationale of this delay is to allow an adequate recovery of tissues from surgical trauma and to minimize the risk of persistent urinary incontinence.

RP is performed according to the recommended technique for locally advanced PC [25]. The additional time required for IOERT is short, on average 30 minutes [12–14, 19].

In the Italian IOERT studies there are no significant differences in terms of surgical complications, early toxicity, 1-year continence rate, and late side effects [12–14, 19]. No major surgical complications were described by all authors. Rocco et al. reported higher blood loss and need of transfusion for IOERT patients compared to those treated by conventional RP. However, this difference was not statistically significant (42% versus 30%) [19].

IOERT gastrointestinal and genitourinary toxicities are always low and similar to those of EBRT [26, 27]. In Rocco's paper, a comparable toxicity between IOERT + EBRT and EBRT was also reported [19].

Rectal dosimetry showed a mean dose delivered to the anterior rectal wall of 3.5 Gy with a range of 0.44–7.99 [14, 20]. A relevant dose reduction was constantly observed at the level of the posterior rectal wall showing that the rectum was in the steep component of the in-depth dose-distribution curve.

Several questions still remain unsolved. IOERT is part of multidisciplinary approaches for high-risk, locally advanced PC. Therefore, it is difficult to discriminate its contribution to the oncological outcomes. Furthermore, the published series are small and with short follow-up and the optimal IOERT technique is still unclear (IOERT before or after prostate removal, dose of radiation). Current clinical staging is not optimal, and a proportion of patients are at risk of overtreatment when IOERT is delivered (about 1/3 of the patients in the literature series had negative surgical margins and pT2 disease).

## 5. Conclusion

IOERT is safe and feasible with a low complication rate after short-intermediate follow-up. Combined RP and IOERT are potentially an effective first step in the multimodality approach for the treatment of high-risk PC. Finally, comparative trials are needed to allow a statistically powerful comparison of IOERT outcomes with those of gold standard treatments for high-risk PC. Until long-term safety and oncological results of IOERT are not available, this technique should be considered an experimental option in the treatment of high-risk PC.

## Figures and Tables

**Table 1:** Treatment modality and outcomes of selected series of IORT in prostate cancer.

	pts	Treatment modality	Local control	Overall survival (5 years)	Morbidity, toxicity, and surgical complications
Takahashi et al. [9]	9	IORT(20–25 Gy) + PNLD + EBRT(50 Gy) ± HT—without RP	100% 5y	—	No severe toxicity
	5	IORT(30–35 Gy) + PNLD ± HT—without RP	80% 5y		No severe toxicity

Abe et al. [17]	21	IORT (28–35 Gy) single dose + PNLD—without RP IORT(20–25 Gy) + EBRT(50 Gy) ± HT	81% 5y	72%	100% hematuria

Kojima et al. [10]	30	IORT(12–20 Gy) + PRP/RRP ± PNLD + EBRT ± HT	—	43%	—

Higashi et al. [11]	35	PNLD + IORT(25–30 Gy) + EBRT(30 Gy) ± HT—without RP	—	92% (stage B)	No severe toxicity
				87% (stage C)	

Saracino et al. [12]	34	IORT(16–22 Gy) + RRP ± PNLD + EBRT ± HT	91%	—	No severe toxicity

Rocco et al. [19]	33	RRP + PNLD + IORT(12 Gy) + EBRT ± HT	—	—	1 lymphocele 3 anastomotic stricture

Krengli et al. [14, 20]	38	RRP + PNLD + IORT(9–12 Gy) + EBRT ± HT	—	—	11% G2 GE toxicity
					4% G2 GU toxicity
					5 lymphocele
					2 pelvic hematoma

RP = radical prostatectomy, PRP = perineal radical prostatectomy, RRP = retropubic radical prostatectomy, PLND = pelvic lymphadenectomy, EBRT = external-beam radiation, HT = hormonal therapy, GE = gastro-enteric, GU = genito-urinary.
